# Effects of recumbent isometric yoga on the orthostatic cardiovascular response of patients with myalgic encephalomyelitis/chronic fatigue syndrome

**DOI:** 10.1186/s13030-025-00336-w

**Published:** 2025-09-01

**Authors:** Takakazu Oka, Battuvshin Lkhagvasuren

**Affiliations:** 1 Department of Psychosomatic Medicine, International University of Health and Welfare Hospital, Iguchi 537-3, Nasushiobara, Tochigi, 329-2763 Japan; 2 Department of Psychosomatic Medicine, Kyushu University Hospital, Maidashi 3-1-1, Fukuoka, Higashi-Ku, 812-8582 Japan

**Keywords:** Myalgic encephalomyelitis/chronic fatigue syndrome, Isometric yoga, Postural orthostatic tachycardia syndrome, Orthostatic intolerance, Treatment

## Abstract

**Background:**

Our previous studies demonstrated that the regular practice of recumbent isometric yoga reduced the fatigue of patients with myalgic encephalomyelitis/chronic fatigue syndrome (ME/CFS). Some patients with ME/CFS have postural orthostatic tachycardia syndrome (POTS); however, the effects of recumbent isometric yoga on orthostatic cardiovascular responses and whether recumbent isometric yoga improves POTS remain unknown. This pilot study was done to investigate the effect of recumbent isometric yoga on the orthostatic cardiovascular response of patients with ME/CFS.

**Main body:**

Ten adult female patients with ME/CFS performed recumbent isometric yoga for 12 weeks. Changes in their systolic blood pressure (SBP), diastolic blood pressure (DBP), and the pulse rate (PR) during an active standing test were compared before and after the 12-week regimen. Among the 10 patients, 8 manifested a normal orthostatic response and 2 manifested POTS before the yoga intervention. Patients who manifested a normal orthostatic response before yoga also manifested the normal orthostatic pattern after the yoga intervention. In contrast, the two patients who manifested POTS before the regimen showed a normal orthostatic response after completing the yoga intervention.

**Conclusions:**

This study found that the patients who manifested POTS and performed recumbent isometric yoga for 12 weeks had a reduced increase in PR after standing up. This pilot study suggests that recumbent isometric yoga would be useful as an adjunctive nonpharmacological intervention for improving POTS in patients with ME/CFS. This finding should be confirmed in a larger number of cases.

## Background

Myalgic encephalomyelitis/chronic fatigue syndrome (ME/CFS) is a debilitating disease characterized by persistent or relapsing unexplained fatigue of at least six months duration that is not relieved by rest and causes a substantial reduction in daily activities [1, 2]. Some patients are so ill that they spend most of their day in bed. To date, however, there is no established treatment for these patients. Therefore, based on our previous study that demonstrated that practicing seated isometric yoga reduced the fatigue of patients who could sit for at least 30 min [3], we developed a recumbent isometric yoga program so that even severely ill patients can practice to reduce their fatigue [4]. It is a 30-min program that patients can practice on their bed in a recumbent position. It consists of three parts: (1) adjusting external and internal conditions; (2) relaxation of excessive lumbar lordosis followed by isometric poses of the neck, shoulders, lower back, hip, elbows, and heels; and (3) deep relaxation or Savasana. Previous studies have demonstrated that the regular practice of a recumbent isometric yoga program reduced fatigue without exacerbating patient symptoms or inducing postexertional malaise (PEM) in patients with ME/CFS who were almost bedridden [4, 5]. 

In addition to fatigue, many patients with ME/CFS exhibit orthostatic intolerance, with 11–27% of ME/CFS patients manifesting postural orthostatic tachycardia syndrome (POTS) [6, 7]. POTS is a form of orthostatic intolerance that is characterized in adults by an increased pulse rate (PR) upon standing of 30 beats/min (bpm) or more in the absence of orthostatic hypotension [8]. This paper was written to determine the effects of recumbent isometric yoga on orthostatic cardiovascular responses and whether recumbent isometric yoga improves POTS, which are currently unknown, through the analysis of changes in systolic blood pressures (SBPs), diastolic blood pressures (DBPs), and PRs during an active standing test before and after a 12-week intervention of recumbent isometric yoga.

## Methods

### Subjects

Ten adult female patients with ME/CFS (aged 36.5 ± 12.2 years) who had been treated in the department of psychosomatic medicine and did not obtain complete recovery participated in the study. The patients were diagnosed with ME/CFS based on the following diagnostic criteria: the 1994 Fukuda case definition of CFS [1], the 2003 Canadian clinical case definition of ME/CFS [2], the 2011 International Consensus Criteria for ME [9], the 2015 case definition of systemic exertion intolerance disease (SEID) [10], and the 2017 clinical case definition of ME/CFS in Japan [11].

### Methods

The participants performed an active standing test before and after undergoing a 12-week recumbent yoga regimen. The test was conducted in the afternoon between 2 and 4 p.m. On the test day, each participant confirmed that she had not smoked or consumed caffeine on that day. After sufficient rest, the participant lay in a supine position for 10 min, which was followed by a standing position for 7 min. The SBP, DBP, and PR were recorded every minute by an electric sphygmomanometer (Nico PS501; Parama-tech, Japan). The baseline SBP, DBP, and PR were defined as the last value before standing. The change in PR (△PR) was defined as the difference between the maximal PR after standing and the baseline value. The patients also answered the Japanese version of the 11-item Chalder Fatigue Scale (CFQ 11) questionnaire, which is a well validated, self-reported Likert scale that measures the severity of fatigue in patients with ME/CFS. The maximum score is 33 [12, 13].

### Yoga intervention

Patients practiced a recumbent isometric yoga program for 12 weeks. The program takes 30-min to perform, but can be modified according to the treating physician’s and yoga instructor’s observations of a patient’s condition. While practicing is on a one-on-one basis with a yoga instructor during visits to the hospital, at home each patient practiced it with the aid of a video shown on YouTube (Isometric Yoga Program for ME/CFS and Long COVID) [14]. In brief, in a quiet, dimly lit, and comfortable room, the patients performed several isometric poses very slowly while breathing, with coordinated matched voicing or unvoiced, while using 30%–50% of their maximal muscular strength, followed by Savasana. For some patients, the number of repetitions per pose was reduced or some poses were omitted depending on their physical condition. After each session, they were asked to generalize this relaxed and peaceful state to their daily lives to improve their ability to cope with fatigue. The details of the poses and medical background behind the procedures were previously described elsewhere [3–5, 15, 16]. Adverse events and patient adherence were also monitored, as previously described [4]. Each patient practiced approximately four times/week. During the intervention period, the participants continued with their pharmacotherapy, with unchanged doses of prescribed medications.

### Statistical analysis

Data are presented as means ± standard deviation (SD). Differences between the mean scores of outcome measures were tested by two-way analysis of variance, with measurement repetition. SPSS for Windows, V.21 software was used for statistical analysis.

## Results

**Fig. 1:**
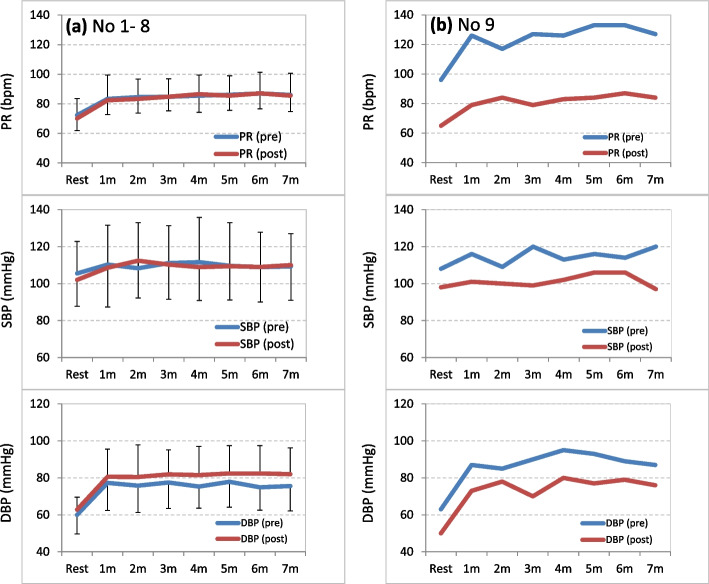
Effects of recumbent isometric yoga on cardiovascular response to standing. Changes in the PR, SBP, and DBP of (a) patients 1–8 (means ± SD) and (b) patient number 9. PR, SBP, and DBP were measured with the patient lying down (“resting”) and at 1, 2, 3, 4, 5, 6, and 7 min after standing. Pre, pre-intervention; post, post-intervention; m, minutes; PR, pulse rate; SBP, systolic blood pressure; DBP, diastolic blood pressure

Among the 10 patients, 8 (patient #’s 1–8) exhibited a normal orthostatic pattern and 2 (patient #’s 9 and 10) exhibited POTS before the yoga intervention (Table 1).  Patients with a normal orthostatic response before the intervention also exhibited a normal orthostatic pattern after the intervention. Their SBP, DBP, and PR were not different before and after they completed the yoga intervention. In contrast, the two patients who showed POTS before the intervention demonstrated a normal orthostatic pattern after the intervention; i.e., the △PR changed from 37 to 22 bpm and from 40 to 29 bpm in patient #’s 9 and 10, respectively. Both patients reported light-headedness when standing up before the yoga intervention but did not feel light-headed after the intervention. Changes in the SBP, DBP, and PR during the standing test of the normal patients and patient #9 pre- and postintervention are illustrated in Fig. 1. None of the patients reported any serious adverse events associated with practicing recumbent isometric yoga.

**Table 1:** Demographic characteristics of the patients

Group number	Age	Chalder FS score	SBP (mmHg)	DBP (mmHg)	PR (bpm)	△PR (bpm)
Normal						
1–8	37.8 ± 13.2	22.1 ± 7.2	105.5 ± 17.3	60.0 ± 6.8	72.3 ± 11.2	15.9 ± 5.3
POTS						
9 10	26 37	27 32	108 93	63 74	96 68	37 40

Chalder FS, SBP, DBP, PR at baseline, and △PR are values at the pre-intervention period. In the normal group, mean values (mean ± standard deviation) of patient numbers 1 to 8 are shown. In the POTS group, individual values of patient numbers 9 and 10 are shown

△PR = maximal PR during the standing test – baseline PR

*DBP* diastolic blood pressure, *FS* fatigue scale, *POTS* postural orthostatic tachycardia syndrome, *PR* pulse rate, *SBP* systolic blood pressure

## Discussion

This study demonstrated that patients with a normal orthostatic response who practiced recumbent isometric yoga for 12 weeks did not show changes in their SBP, DBP or PR; however, the patients with POTS showed improvement after practicing recumbent isometric yoga. The results showed that recumbent isometric yoga was beneficial for these patients with ME/CFS and POTS. To our best knowledge, this is the first study that has investigated the effects of recumbent isometric yoga on orthostatic cardiovascular response.

Currently, yoga is widely recognized and practiced as a mind–body therapy. After considering the characteristics of ME/CFS and the limitations of patients with this illness, we developed isometric yoga programs to improve the fatigue of patients with ME/CFS without inducing postexertional malaise (PEM) [3, 4]. Our previous studies have suggested that postisometric muscular relaxation [17, 18], anti-inflammatory effects [15, 16], and improvement in negative affect such as depression [16] that were induced by the regular practice of isometric yoga may contribute to improvement of the fatigue of ME/CFS patients.

When we developed the isometric yoga program, we intentionally excluded standing postures to avoid the development of POTS [3, 4]. However, to our surprise, this study demonstrated that recumbent isometric yoga led to its amelioration in the two patients who had manifested it. Both patients with POTS were seriously ill, with Chalder FS scores higher than the scores of the patients showing a normal orthostatic response, and both had been bedridden for a long time, strongly suggesting that they were deconditioned. It is well known that deconditioning is one of the causes of POTS [19–21]. Recent studies have suggested the usefulness of physical exercise to treat POTS [22]. However, it is difficult to introduce exercise therapy for patients with ME/CFS, because they rapidly become exhausted and develop PEM after minimal physical exertion [23].

The recumbent isometric yoga practice entails isometric muscle contractions that involve the lower limbs. While practicing, patients can easily adjust muscular resistance according to their condition during the time of practice. This type of exercise might counteract deconditioning while leading to the amelioration of POTS in patients with ME/CFS without resulting in PEM. Thus, the results of this study suggest that recumbent isometric yoga would be a promising exercise therapy for improving deconditioning and POTS in patients who have difficulty with exercises such as walking.

When we developed this program, we also hypothesized that practicing an isometric yoga program would have cognitive and behavioral benefits, such as increasing awareness of inner sensations by slowly performing each pose while paying mindful attention to breathing and movement and enabling more therapeutic coping behaviors, such as appropriate pacing, based on yoga-induced interoceptive awareness [3–5]. To date, it isn’t known how interoceptive training affects POTS-related symptoms. However, it is possible that the cognitive-behavioral changes or increased interoceptive awareness induced by yoga may have a physiological effect that leads to the improvement of POTS-related symptoms such as lightheadedness. This hypothesis is supported by previous studies suggesting that sustained focus on changing bodily sensations enabled patients to effectively utilize mindfulness-related skills, re-establishing sensorimotor integration and facilitating non-judgmental reconnection with bodily sensations, which leads to improvements in physical and psychological symptoms, functionality, and quality of life for patients with fibromyalgia [24, 25], a common comorbidity of ME/CFS.

This study has several limitations. First, the number of participants was small, and the findings will need to be confirmed in a larger study. Second, patients were already undergoing pharmacotherapy when the intervention started. Although none of the patients received β-blockers, some patients were receiving α1-adrenoceptor agonists to improve their orthostatic intolerance. Therefore, it is unclear whether recumbent isometric yoga would be effective for patients who are not receiving pharmacotherapy. Despite the limitations, to our best knowledge this study for the first time has demonstrated the possibility that recumbent isometric yoga would be useful as an adjunct, nonpharmacological, intervention for ameliorating POTS in patients with ME/CFS.

## Conclusions

This study demonstrated that the practice of recumbent isometric yoga for 12 weeks in ME/CFS patients with POTS reduced their PR on standing, while patients with ME/CFS maintained their normal orthostatic response. Although this finding will need to be confirmed in a study with a larger number of participants, the results of this study suggest that recumbent isometric yoga would be useful as a nonpharmacological intervention for improving the POTS of patients with ME/CFS.

## Data Availability

Not applicable.

## Abbreviations

ANOVA: Analysis of variance
bpm: Beats per minute
CFS: Chronic fatigue syndrome
DBP: Diastolic blood pressure
FS: Fatigue scale
ME: Myalgic encephalomyelitis
PEM: Postexertional malaise
POST: Postural orthostatic tachycardia syndrome
PR: Pulse rate
SBP: Systolic blood pressure
SD: Standard deviation
